# Dual-Target Peptide-Modified Erythrocyte Membrane-Enveloped PLGA Nanoparticles for the Treatment of Glioma

**DOI:** 10.3389/fonc.2020.563938

**Published:** 2020-10-21

**Authors:** Yuexin Cui, Jiejie Sun, Wenyan Hao, Mengyu Chen, Yingzi Wang, Fenghua Xu, Chunsheng Gao

**Affiliations:** ^1^School of Chinese Materia Medica, Beijing University of Chinese Medicine, Beijing, China; ^2^State Key Laboratory of Toxicology and Medical Countermeasures, Beijing Institute of Pharmacology and Toxicology, Beijing, China; ^3^Department of Pharmacy, People's Liberation Army of China (PLA) General Hospital, Beijing, China

**Keywords:** dual-targeting, biomimetic nanoparticles, NGR, ^D^WSW, blood–brain barrier, blood–brain tumor barrier, euphorbia factor L1, glioma

## Abstract

Penetration of the blood–brain barrier (BBB) and the blood–brain tumor barrier (BBTB) remains a significant challenge for the delivery of drugs in the treatment of glioma. Therefore, the development of targeted preparations with the ability to penetrate the BBB and BBTB, and target gliomas, is an important approach if we are to improve the efficacy of glioma treatment. In the current study, an active targeting preparation based on PLGA nanoparticles coated with erythrocyte membranes (RBCNPs) and dual-modified with ^D^WSW and NGR peptide ligands (^D^WSW/NGR-RBCNPs). Euphorbia factor L1 (EFL1) extracted from euphorbiae semen was used as the model drug. The final nanoparticles were characterized by *in vivo* and *in vitro* tests. *In vitro* results showed that EFL1-loaded ^D^WSW/NGR-RBCNPs were taken up by cells and had the ability to penetrate the BBB and BBTB and produce cytotoxic effects. Furthermore, *in vivo* studies in mice showed that when injected intravenously, these specialized NPs could enter the brain, target tumor tissue, and significantly extend life span. The results showed that dual-targeting EFL1-loaded ^D^WSW/NGR-RBCNPs have significant potential as a nanotherapeutic tool for the treatment of brain glioma.

## Introduction

Biomimetics has been recognized as an important mission in science and engineering for a long time. Currently developed drug delivery carrier materials are usually synthetic polymer compounds. The carrier materials or the carrier surface are modified according to the different purposes of use (1–4). However, such modification often fails to fully recognize complex endogenous substances in the body. And sometimes it is even regarded as exogenous poisons excreted from the body, failing to reach the lesion site and achieve the desired effect as designed. Cell membrane biomimetic nanoparticles are realized by using natural cell membrane as the shell to encapsulate the synthesized nanoparticles. Through this strategy, the structure and function of the cell membrane, especially the specific functional proteins on the cell membrane surface, are preserved (2, 5, 6). This means it can also reduce the elimination of nanoparticles.

Glioma is a fatal disease that has high incidence, recurrence, and mortality rates. Clinical data demonstrates that the cure rates for this disease are low (7). The current methods that are commonly used for the treatment of glioma are surgery, radiotherapy, and chemotherapy. However, the clinical efficacy of the drugs used to treat glioma is not satisfactory. Many drugs cannot be used in patients with glioma because of their poor physicochemical properties, lack of targeting capability, and their inability to penetrate the blood–brain barrier (BBB) and blood−brain tumor barrier (BBTB) (8–10). Therefore, there is significant interest in developing new ways to deliver therapeutic drugs to the site of gliomas.

Dual-targeting nanocarriers have already demonstrated their ability to circumvent the BBB and BBTB and deliver specific drugs to glioma sites (11). Over the past few decades, a range of nanocarriers have emerged; many of these exploit different materials to alter their properties. However, these synthetic materials are generally associated with poor efficacy and commonly cause toxicity (12, 13). Research has shown that many nanoparticles are eliminated by the immune system, thus resulting in a reduction in therapeutic effect (14–17). Over recent years, the membranes of erythrocytes have attracted significant attention within the nanotechnology sector, largely because of they are easily obtained and their low levels of immunogenicity. Encapsulating erythrocyte/red blood cell membranes on poly(lactic-co-glycolic acid) (PLGA) nanoparticles (RBCNPs) with targeting modifications has already been shown to achieve unexpected therapeutic effects (1, 2, 18–20).

Typically, the surface of the erythrocyte membrane is modified with a peptide by lipid-insertion. Quorum sensing (QS) signaling molecules are one of them. Coordinated changes in a growing microbial population that are achieved through signaling molecules is referred to as quorum sensing (QS). Some peptides can selectively penetrate the BBB. For example, the ^D^WSW (^D^W^D^S^D^W^D^G^D^P^D^Y^D^S) peptide originates from Clostridium acetobutylicum and could be used to target the brain (21, 22). In addition, specific drugs can be designed in such a way that they can target highly expressed targets in glioma cells. CD13 is one such target and can be targeted by NGR (Asn-Gly-Arg), a peptide that exhibits high affinity for CD13. The CD13 receptor also plays a crucial role in promoting angiogenesis in receptor-mediated anti-angiogenic therapy. Over recent years, ^D^WSW- and NGR-modified nanocarriers have been developed (23–26).

Euphorbia factor L1 (EFL1) is a lathyrane-type diterpenoid active component that is extracted and separated from Euphorbiae Semen (seeds of *Euphorbia lathyris* L.), a traditional form of Chinese medicine. Existing research shows that EFL1 has significant antitumor effects and has been demonstrated to exert cytotoxic effects in HeLa, A549, C6, MCF-7, and HL-60 cells; it can also reverse activity against P-glycoprotein-mediated multidrug resistance in cells (27–31). However, the clinical application of EFL1 is limited by its poor water solubility and low bioavailability. The solubility of EFL1 can be increased by encapsulation in PLGA nanoparticles (32, 33).

**Figure 1:**
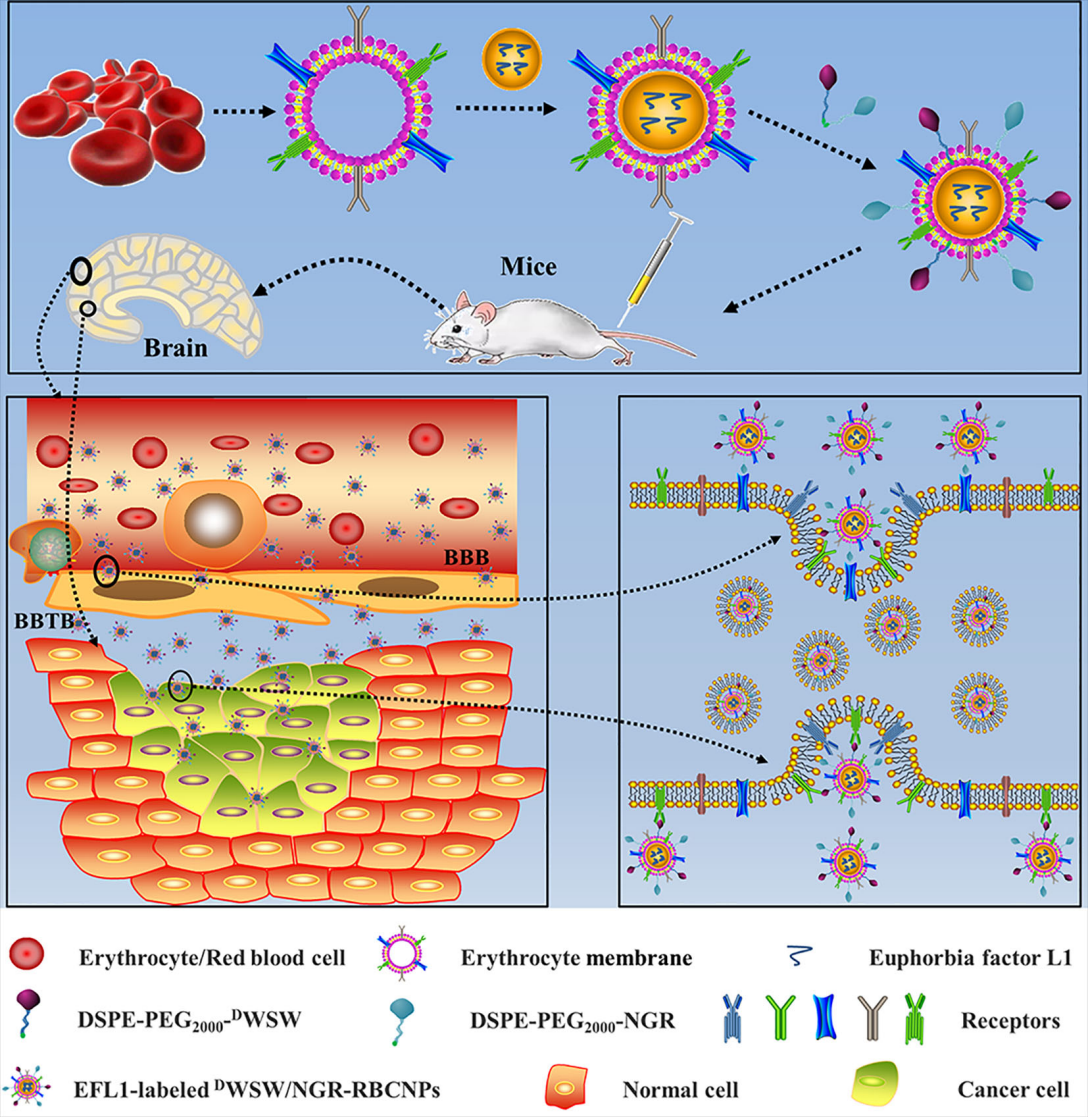
Graphical abstract of this study. Schematic illustration of dual-target peptides modified using erythrocyte membrane-enveloped PLGA nanoparticles for the treatment of glioma. The nanoparticles were designed to penetrate the BBB and BBTB and then to aggregate at tumor sites. ^D^WSW peptide was used to penetrate BBB and NGR was used to target tumor. ^D^WSW/NGR-RBCNPs were observed to selectively accumulate in tumor tissue and exert a therapeutic effect.

Based on these previous findings, we designed dual-targeting RBCNPs that were modified with ^D^WSW and NGR by lipid insertion. These nanocarriers were able to penetrate the BBB and the BBTB synergistically and target glioma cells. The efficacy of these dual-modified RBCNPs (^D^WSW/NGR-RBCNPs) following EFL1 encapsulation with respect to glioma treatment was evaluated using both *in vitro* and *in vivo* approaches (**Figure 1**). The current study aimed to develop a safe and effective treatment for glioma.

## Materials and Methods

### Materials

PLGA, with a lactic/glycolic acid ratio of 75/25 (5600 Da), was obtained from Jinan Daigang Biomaterial Co., Ltd (Shandong, China). 1,2-distearoyl-sn-glycero-3-phosphoethanolamine -N-[maleimide(polyethylene glycol)-2000] (DSPE-PEG_2000_-Mal), ^D^WSW, and NGR peptides were purchased from Xi`an ruixi Biological Technology Co., Ltd (Shanxi, China). Euphorbia factor L1 (EFL1) was obtained from Chendu Dsiter Co., Ltd (Sichuan, China). Anti-CD47 antibodies were purchased from Abcam (Cambridge, UK). All chemical reagents were of analytical grade and were purchased from Macklin Biochemical Co., Ltd (Shanghai, China).

### Cells and Experimental Animals

Glioma cells (C6), mouse brain endothelial cells (bEnd.3), and human umbilical vascular endothelial cells (HUVECs), were supplied by the Cell Resource Centre of IBMS (Beijing, China) and cultured in Dulbecco’s modified Eagle’s medium (DMEM) containing 10% FBS (Gibco) and 100 IU penicillin.

Female and male ICR mice (initially weighing 18–22 g) were purchased from SPF Biotechnology Co., Ltd (Permit number: SCXK (Jing) 2019-0010, Beijing, China). All animal experiments complied with the National Laboratory Animal Management Regulations, and the Beijing Municipal Laboratory Animal Management Regulations. All procedures and experiments involving the care and handling of animals were carried out with the approval of the Animal Care and Use Ethics Committee of Beijing Institute of Pharmacology and Toxicology (Beijing, China).

### Preparation of Peptide-Modified RBCNPs

#### Synthesis and Characterization of Materials

DSPE-PEG_2000_-^D^WSW and DSPE-PEG_2000_-NGR were synthesized using the sulfhydryl-maleimide coupling method between thiolated peptides and DSPE-PEG_2000_-Mal. In brief, DSPE-PEG_2000_-Mal in PBS (pH 7.4) was slowly added to a ^D^WSW or NGR (1:1 molar ratio) solution (PBS, pH 7.4) at room temperature for 24 h while stirring. Unreacted compounds were then removed by washing with distilled water (MWCO 3.0 kDa). The final solution was freeze-dried to await further use. Prior to application, the compounds were assessed by MALDI-TOF mass spectrometry (MALDI-TOF MS).

#### Collection of Erythrocyte Membranes

Erythrocyte membranes were collected as previously reported (34, 35). In brief, cells were collected from mice and transferred into tubes coated with an anticoagulant. Plasma and leukocytes were removed by centrifugation (3,000 rpm for 10 min at 4°C). PBS (pH = 7.4) was then added into the tubes and centrifuged at 3,000 rpm for 10 min at 4°C; this procedure was repeated three times. The red blood cells (RBCs) were suspended in 0.25 × PBS (pH = 7.4), stored at 4°C for 0.5 h, and then centrifuged (8000 rpm for 10 min at 4°C); this procedure was repeated three times. Finally, the erythrocyte membranes were suspended in PBS (pH = 7.4) and stored at 4°C to await further use.

#### Preparation of PLGA Nanoparticles

PLGA nanoparticles (NPs) were prepared by a nano-precipitation method, as described previously (36–38). A total of 40 mg of PLGA, and 4 mg of EFL1 or Cy5.5 (hydrophobic probe, 100 μL), was added to 4 mL of acetone. Once completely dissolved, the mixture was poured into 10 mL of water slowly. Then, the mixture was then stirred in open air for 24 h to eliminate the organic solvent; the resultant products were EFL1-loaded NPs or Cy5.5-labelled NPs.

#### Preparation of RBCNPs Modified With Peptides

Erythrocyte membranes were sonicated for 3 min in a bath sonicator (KQ3200, Kunshan, China) at a frequency of 37 kHz and a power of 250 W. Using a mini-extruder (Avanti Polar Lipids, AL, USA), we isolated erythrocyte membrane vesicles by extruding the membranes repeatedly through 400 nm and 200 nm polycarbonate porous membranes.

To prepare nonmodified-RBCNPs (N-RBCNPs), EFL1-labelled NPs or Cy5.5-loaded NPs were mixed with erythrocyte membrane vesicles (at a ratio of 3:1) and then extruded through a 200 nm polycarbonate membrane at least 10 times; this procedure created EFL1-loaded RBCNPs or Cy5.5-labelled RBCNPs. The ^D^WSW-modified RBCNPs (^D^WSW-RBCNPs), NGR-modified RBCNPs (NGR-RBCNPs), and ^D^WSW/NGR modified RBCNPs (^D^WSW/NGR-RBCNPs), were prepared using a lipid-insertion technique. DSPE-PEG_2000_-^D^WSW (4%, weight ratio of DSPE-PEG_2000_-^D^WSW to NPs), or DSPE-PEG_2000_-NGR (3%, weight ratio), was added to RBCNPs in PBS (pH 7.4) at 37°C with stirring for approximately 0.5 h. In order to prepare ^D^WSW/NGR-RBCNPs, both DSPE-PEG_2000_-^D^WSW and DSPE-PEG_2000_-NGR were added to the solution.

### Physicochemical Characterization of Dual-Modified RBCNPs

#### Binding Efficiency of Peptides Onto RBCNPs

We created a DSPE-PEG_2000_-peptide-fluorescent probe by reacting the carboxyl group in a fluorescent probe with the primary amine group in the DSPE-PEG_2000_-peptide. Specifically, DSPE-PEG_2000_-NGR was labelled with 5-(and 6)-carboxyfluoresceindiacetate (CFDA) using the following protocol. First, CFDA was dissolved in dimethyl sulfoxide; we then added N, N’-dicyclohexyl carbodiimide and N-hydroxysuccinimide (4:2:1). This solution was stirred in the dark for 24 h at room temperature and then centrifuged (4,000 r/min, 15 min) to obtain a supernatant. Next, 1.2% of DSPE-PEG_2000_-NGR, and one drop of triethylamine, were added and allowed to react for 24 h in the dark. Then, the products were dialyzed (MWCO 3.5 kDa) in purified water for 24 h in a light-proof environment. The purified solid product (DSPE-PEG_2000_-NGR-CFDA) was obtained by freeze-drying. Using the same methodology, we labelled DSPE-PEG_2000_-^D^WSW with 5-carboxy-X-rhodamine (5-ROX). The purified solid product (DSPE-PEG_2000_-^D^WSW-5-ROX) was also obtained by freeze-drying. The preparation of fluorescent probe-labelled dual-modified RBCNPs (^D^WSW-5-ROX/CFDA-NGR-RBCNPs) involved DSPE-PEG_2000_-^D^WSW-5-ROX and DSPE-PEG_2000_-NGR-CFDA; the methodology was the same as that used to prepare the ^D^WSW/NGR-RBCNPs.

The DSPE-PEG_2000_-NGR-CFDA, DSPE-PEG_2000_-^D^WSW-5-ROX, or N-RBCNPs, were dispersed in PBS (pH 7.4). Next, the maximum absorption wavelengths of DSPE-PEG_2000_-NGR-CFDA and DSPE-PEG_2000_-^D^WSW-5-ROX were determined using a UV-visible spectrophotometer (scanned at 200-800 nm). The absorbance (A) of CFDA was measured at 493 nm while that of 5-ROX was measured at 578 nm (standard solution, 0.5 to 3.50 μg/mL); this allowed us to perform linear regression analysis.

^D^WSW-5-ROX/NGR-CFDA-RBCNPs were diluted with PBS to measure the total absorbance (*A_Total_*). Then, the absorbance of the free targeting-peptide (*A_Free_*) was determined by ultrafiltration centrifugation (MWCO 300 kDa, 12000 r/min). The formula used to determine the connection efficiency of ^D^WSW-5-ROX/NGR-CFDA-RBCNPs is given in Equation (1).

(1) $$\text{Connection efficiency}=(A_{Total}-A_{Free})/A_{Total}\times 100\%$$

#### Characterization of Dual-Modified RBCNPs

Biomimetic nanoparticle particle size distribution and zeta potential were measured by dynamic light scattering (Litesizer™ 500, Anton-Paar, Austria). Transmission electron microscopy (TEM) (HITACHI, H-7650, Japan) was used to characterize the morphology of EFL1-loaded ^D^WSW/NGR-RBCNPs. Protein analysis was performed by sodium dodecyl sulfate-polyacrylamide gel electrophoresis (SDS-PAGE) (39). Protein concentration was determined using a BCA assay kit (Pierce, China) and western blotting was used to analyze the expression levels of the glycoprotein CD47 (40). Turbiscan Lab^®^ Expert (Formulaction, L’Union, France) was used to evaluate the 72-hour stability of EFL1-loaded ^D^WSW/NGR-RBCNPs in FBS (37°C); we also used this instrument’s software to apply a variety of back-scattering (ΔBS) profiles to investigate stability.

Equation (2) and (3) was used to calculate the EFL1 encapsulation efficiency (EE) and drug loading capacity (DL) for various particles.

(2) $$\text{EE}\%=(W_{total\;drug}-W_{free\;drug})/W_{total\;drug}\times 100\%$$

(3) $$\text{DL}\%=(W_{total\;drug}-W_{free\;drug})/W_{total\;drug\;and\;carriers}\times 100\%$$

*W_total drug_* represents the total drug in the NPs, *W_free drug_* represents the amount of free drug removed by ultrafiltration while *W_total drug and carriers_* represents the weight of total drug and carriers. The amount of EFL1 was determined by HPLC analysis.

### *In Vitro* Evaluation of Dual-Modified RBCNPs

#### *In Vitro* Release

The release of EFL1 from different nano-preparations was determined using dialysis buffer (pH 7.4). Approximately 1 mL of the various nanoparticle solutions was added to a dialysis bag (MWCO 12 kDa). A release study was then performed in 100 mL of medium at 37°C; 1 mL of medium was removed for analysis at 0, 0.5, 1, 2, 4, 6, 8, 10, 12, 24, 36, and 48 h and was replaced with the same volume of fresh medium. HPLC was then used to determine the concentration of EFL1 released at different times.

#### *In Vitro* Cell Uptake

In order to investigate the uptake of various RBCNPs, we incubated a variety of different cells (C6, bEnd.3, and HUVECs) in 200 μL of DiI-labeled mono- or dual-modified RBCNPs at 37°C for 2 h. Controls were also included in these experiments; these involved nanoparticles that were devoid of ligands. After the experiment, cells were washed and centrifuged three times with cold PBS. We then analyzed the cells qualitatively using confocal laser scanning microscopy (LSM 880, Zeiss, Germany); quantitative analysis was performed by flow cytometry (FCM).

#### *In Vitro* Cytotoxicity Assays

The MTT assay was used to evaluate the cytotoxicity of different nanoparticles in C6 cells. Cells were first seeded in 96-well plates (approximately 5,000 cells per well). Then, cells were exposed to various concentrations of the RBCNP formulations. After 48 h, we added 20 μL of MTT solution (5 mg/mL in PBS) to each well. After a 4 h period of incubation, we determined cell viability at 490 nm using a plate reader (Tecan Spark, Austria).

#### *In Vitro* Transport Across the BBB and BBTB

An *in vitro* BBB model was established as reported previously by Ying Man et al. (41). To summarize, bEnd.3 cells were inoculated at a density of 1.0 × 10^5^ cells per well (Corning, NY, USA). Successful creation of the BBB was confirmed by measuring transendothelial electrical resistance; the BBB transport assay was performed when resistance reached 200 Ω.cm^2^ (42). Different Cy5.5-labeled RBCNPs (50 μM) were then added to DMEM containing 10% FBS for further culture. After 4 h, the solution was collected from the basal chamber and the fluorescence intensity was monitored using a fluorescence spectrophotometer (Agilent Cary Eclipse, USA). HUVECs were seeded in the upper inserts of the transwell and C6 cells were seeded in the lower chamber (at a ratio of 5:1) to create the final *in vitro* BBTB model (43). Different Cy5.5-labeled RBCNPs were then added to the culture medium in each upper chamber. After 4 h, the solution was collected from the lower chamber and the fluorescence intensity was determined by fluorescence spectrophotometry.

#### *In Vitro* Targeting Ability

The *in vitro* BBB model was created by bEnd.3 and C6 cells, as previously reported (43). To summarize, the upper side of a transwell insert was seeded with approximately 1.0 × 10^5^ bEnd.3 cells. The basolateral compartment was then seeded with 2000 C6 cells per compartment. The experiment was performed after 5 d of incubation. Free EFL1, and various EFL1-loaded nanoparticles, were added to the apical compartment. The final concentration of EFL1 in each compartment was 50 μg/mL. Then, 48 h later, the survival rate of the C6 cells was determined with a sulforhodamine-B staining assay (44).

### *In Vivo* Evaluation of Dual-Modified RBCNPs

#### *In Vivo* Glioma Targeting Ability

A glioma-bearing mouse model was established by inoculating C6 cells into the brain, as described previously (45). After 7 d, the brain glioma was evaluated by magnetic resonance imaging (MRI) (PharmaScan 70T/16, Bruke, US). We then selected glioma-bearing mice with similar tumor volumes and measured their basal levels of fluorescence levels prior to treatment. Different RBCNPs were then labeled with DiR for *in vivo* imaging studies. Next, the animals were administered with DiR-loaded N-RBCNPs, DiR-loaded ^D^WSW-RBCNPs, DiR-loaded NGR-RBCNPs, and DiR-loaded ^D^WSW/NGR-RBCNPs, *via* tail vein injection. *In vivo* imaging was carried out 12 h later using a small animal imaging system (IVIS^®^ Spectrum, PerkinElmer, USA) and Living Image^®^ software (Caliper, Alameda, CA) used to quantify bioluminescence and fluorescence signals. Following *in vivo* imaging, we isolated the main organs of each mouse and determined the tissue distribution of each type of nanoparticle. DiI-labeled RBCNPs were then used for brain distribution studies. After 4 h, mice were sacrificed and the brain tissues were harvested. These tissues were then frozen in O.C.T. (Sakura, Torrance, CA, USA), cut into 5 mm frozen sections, stained with DAPI, and analyzed by CLSM.

#### Antitumor Effect in Mice

Glioma-bearing mice were randomly divided into six groups (10 mice per group): a normal saline group, a free EFL1 group, an EFL1-loaded N-RBCNP group, an EFL1-loaded ^D^WSW-RBCNP group, an EFL1-loaded NGR-RBCNP group, and an EFL1-loaded ^D^WSW/NGR-RBCNP group. Seven days after the injection of C6 cells, each mouse received a drug at a dose of 100 μg/kg (based on EFL1 in the formulations); the dose was administered once each day. In each mouse, we then recorded the length of time between tumor cell inoculation and death. Survival times were used to plot Kaplan-Meier survival curves for each group.

#### Histopathological Examination of Brain Tumors

Mice were sacrificed at the end of drug treatment, and their brains were isolated. Each brain was fixed in 10% formalin buffer for 24 h, embedded in paraffin, cut into 5 μm sections, and stained with hematoxylin and eosin. Histopathological examinations were then carried out with a light microscope (Olympus, Japan) with a view to identify tumor tissue and acquire representative digital images.

### Acute Toxicity Evaluation

Mice were randomly divided into three groups (6 mice per group) as follows: a normal saline group, a free EFL1 group, and an EFL1-loaded ^D^WSW/NGR-RBCNP group. Mice were administered with 0.1 mL of drug (equivalent to 100 μg/kg of EFL1) and saline by intravenous injection. After 15 d, we extracted blood from the eyeball to perform routine blood tests and to investigate liver and kidney function. We also removed the main organs for histopathological examination (46, 47).

### Statistical Analysis

Quantitative data are expressed as mean ± standard deviation (SD) unless otherwise indicated. One-way analysis of variance (ANOVA) was used to determine significant differences between different groups, and p<0.05 was considered to indicate statistical significance.

## Results

### Physicochemical Characterization of Dual-Modified RBCNPs

#### Preparation and Characterization of Dual-Modified RBCNPs

**Figure 2:**
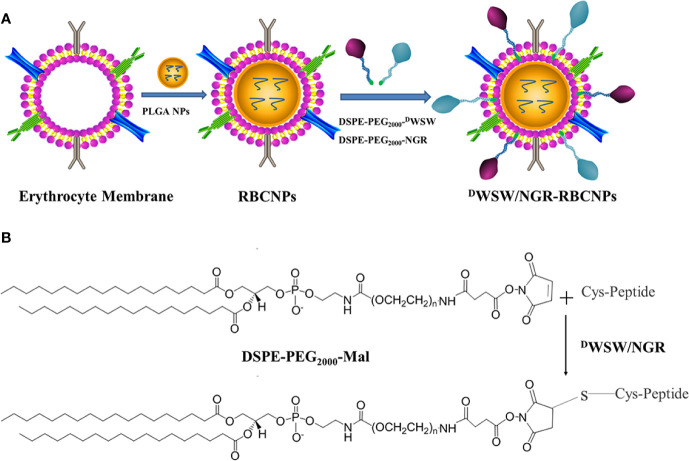
Preparation of ^D^WSW/NGR-RBCNPs. First, the drug was loaded into PLGA, coated with erythrocyte membrane, and finally target-modified **(A)**. The targeting ligand was then synthesized by conjugating DSPE-PEG_2000_-Mal to the cysteine residue on both ^D^WSW and NGR **(B)**. The Michael addition reaction was used in chemical synthesis.

The method used to prepare ^D^WSW/NGR RBCNPs is shown in **Figure 2A**. We synthesized DSPE-PEG_2000_-^D^WSW and DSPE-PEG_2000_-NGR and then prepared double-modified biomimetic PLGA nanoparticles using a lipid-insertion method. **Figure 2B** shows the process used to synthesize the two functional targeting materials. We then coupled the thiol sulfhydryl (-SH) in the ^D^WSW and NGR targeting peptides with DSPE-PEG_2000_-Mal using a sulfhydryl-maleimide reaction. The successful formation of DSPE-PEG_2000_-^D^WSW and DSPE-PEG_2000_-NGR was confirmed by MALDI-TOF MS, with observed mass-charge ratios of approximately 3913 (**Supplementary Figure S1A**) and 3713 (**Supplementary Figure S1B**), respectively; these data confirmed the that the targeted molecular conjugates had been synthesized correctly. The DSPE-PEG_2000_-^D^WSW and DSPE-PEG_2000_-NGR were then used for the target modification of RBCNPs.

**Figure 3:**
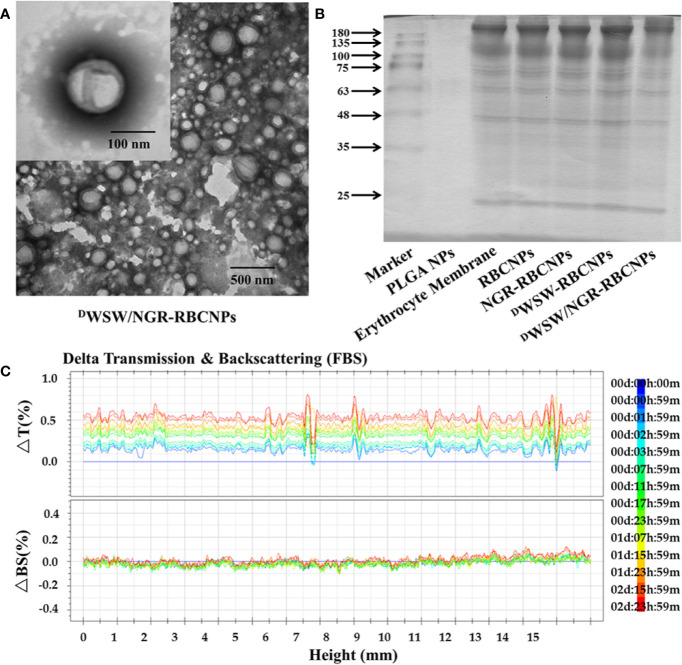
Characterization of nanoparticles. Transmission electron microscopy (TEM) photos of ^D^WSW/NGR-RBCNPs. **(A)** Nanoparticles showing the core-shell structure. **(B)** Erythrocyte membrane proteins in different nanoparticles, showing the protein had not been lost. Similar bands of protein appear in the same location. **(C)** Stability of EFL1-loaded ^D^WSW/NGR-RBCNPs in the presence of FBS; stability fluctuated within a range of 2%. No serious aggregation or sedimentation of the nanoparticles during the measurement period.

After preparation, we used TEM to characterize the morphology of the EFL1-loaded ^D^WSW/NGR-RBCNPs. The typical core-shell structure of EFL1-loaded ^D^WSW/NGR-RBCNPs is shown in **Figure 3A** while **Table 1** summarizes the four distinct RBCNP formulations. The EFL1 encapsulation efficiency (EE) of the erythrocyte membrane-encapsulated nanoparticles was greater than 70%, and the final drug amount was not affected by the targeted peptide modification. The size of nanoparticles is known to be an important determinant of their potential applications *in vivo* and *in vitro*. As shown in **Table 1**, the nanoparticles have a low polydispersity index (DPI) and exhibited a narrow size distribution. It is important that nanoparticles exhibit a uniform size when applied used *in vivo*; physical size is known to influence the therapeutic efficacy of nanoparticles after they have successfully reached the target site (48). In addition, the nanoparticle sizes determined by TEM images (**Figure 3A**) were similar to those determined by a laser particle analyzer (**Supplementary Figure S2B**). Following the encapsulation of erythrocyte membranes, we found that the size of the nanoparticles increased (**Supplementary Figures S2A, B**) while the zeta potential decreased (**Supplementary Figures S2C, D**). The zeta potential of the formulations was important as this ensured that the nanoparticles remained stable in solution.

**Table 1:** Characterization of different nanoparticles.

Sample	Diameter (nm)	PDI	EE (%)	DL (%)
PLGA NPs	129.84 ± 0.48	0.067 ± 0.004	82.95 ± 1.03	2.3 ± 0.4
RBCNPs-EFL1	144.37 ± 1.57	0.106 ± 0.011	77.36 ± 1.12	2.1 ± 0.2
^D^WSW-RBCNPs-EFL1	146.85 ± 1.45	0.116 ± 0.017	74.87 ± 1.45	2.0 ± 0.1
NGR-RBCNPs-EFL1	145.67 ± 1.88	0.119 ± 0.011	76.36 ± 1.17	2.0 ± 0.3
^D^WSW/NGR-RBCNPs-EFL1	148.48 ± 1.20	0.082 ± 0.014	73.65 ± 1.07	1.9 ± 0.1

Data are represented as mean ± SD, n = 3.

Analysis of the protein content of the ^D^WSW/NGR-RBCNPs was carried out to confirm successful functionalization of the nanoparticles with erythrocyte membrane antigens. The proteins in erythrocyte membrane-encapsulated nanoparticles and erythrocyte membranes were assayed in parallel through BCA assay kit. The protein concentration of erythrocyte membrane was 309.0 ± 2.52 μg/mL, and that of double-targeted modified nanoparticles was 244.6 ± 8.22 μg/mL. Considering the dilution of the erythrocyte membrane, the process of erythrocyte membrane-encapsulated nanoparticle formation and targeted modification did not lead to erythrocyte membrane protein loss. The proteins in erythrocyte membrane-encapsulated nanoparticles, and erythrocyte membranes, were assayed in parallel by SDS-PAGE. Neither the formation of erythrocyte membrane-encapsulated nanoparticles, or targeted modification, led to the significant loss of erythrocyte membrane proteins (**Figure 3B**). It was evident that the erythrocyte membrane-encapsulated nanoparticles retained the properties of erythrocyte membranes.

The *in vivo* application of EFL1-loaded ^D^WSW/NGR-RBCNPs requires that they are stable under physiological conditions. Therefore, we used fetal calf serum (FBS) to test the stability of the nanoparticles in blood. We used the Turbiscan Lab^®^ Expert system to evaluate the *in vitro* stability of the EFL1-loaded ^D^WSW/NGR RBCNPs in serum at 37°C for 72 h. This methodology has been reported previously (49), and in the present study indicated that EFL1-loaded ^D^WSW/NGR RBCNPs showed no significant aggregation or sedimentation (transmission or back-scattering profiles of less than 2.0%) in FBS (**Figure 3C**). Increasing evidence has shown that the combined action of multiple membrane components on the cell surface ensures that RBCs are not engulfed by macrophages. In particular, CD47, a protein marker of the erythrocyte membrane, has been shown to inhibit macrophage phagocytosis by interacting with SIRP-α receptors (50). In order to confirm the presence of CD47 on the ^D^WSW/NGR-RBCNPs, we carried out western blotting analysis on a series of distinct samples (NPs, RBC lysate, erythrocyte membranes, and ^D^WSW/NGR-RBCNPs). As shown in **Supplementary Figure S3A**, the results showed that after the process of nanoparticle synthesis and modification had been completed, CD47 was still present on the surface of the RBCNPs. This suggests that nanoparticles coated with erythrocyte membranes can circulate for longer durations in the body and are subject to reduced levels of phagocytosis.

Next, we carried out *in vitro* release studies to investigate the release characteristics of EFL1 in the RBCNPs. There was no significant difference in the EFL1 release behavior of the biomimetic nanocarriers in buffer solution (PH=7.4, **Supplementary Figure S3B**). This finding indicated that targeted modification did not affect drug release.

#### The Efficiency of Connection Between Targeting Peptides and RBCNPs

We used a simple method to investigate the connection efficiency between ^D^WSW and NGR peptides and RBCNPs. First, the two targeting conjugates were reacted with CFDA and 5-ROX to obtain DSPE-PEG_2000_-NGR-CFDA and DSPE-PEG_2000_-^D^WSW-5-ROX, respectively. The maximum UV absorption of DSPE-PEG_2000_-NGR-CFDA was 493 nm while that of DSPE-PEG_2000_-^D^WSW-5-ROX was 578 nm. A mixture of the two peptides could be measured at different wavelengths. Unmodified RBCNPs did not interfere with such measurements (**Supplementary Figure S3C**). Using this method, we were able to ascertain that the connection efficiencies of DSPE-PEG_2000_-NGR and DSPE-PEG_2000_-^D^WSW in the double-modified RBCNPs were 71.27% and 73.45%, respectively.

### *In Vitro* Evaluation

#### Cellular Uptake

Cellular uptake experiments were performed to investigate the affinity of the different types of modified RBCNPs to specific cells. To do this, we incubated bEnd.3, HUVECs, and C6 cells, with different types of DiI-labeled RBCNPs for 2 h at 37°C. A previous study used bEnd.3 cells as a model of the BBB in order to study the penetrating ability of ^D^WSW (51). HUVECs have also been used previously as a model cell for tumor angiogenesis and thus confirm the ability of NGR to target the neovasculature (52).

**Figure 4:**
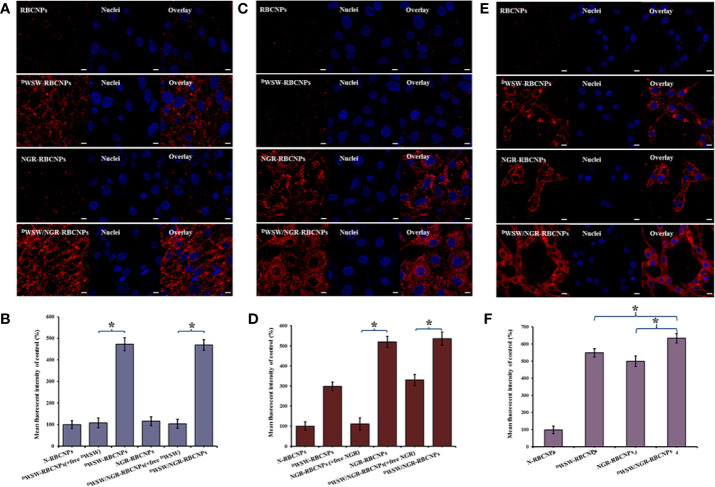
Targeting ability test with different cells. Cellular uptake of different DiI-labelled RBCNPs by bEND.3 cells **(A, B)**. ^D^WSW exhibited the ability to cross the BBB and the free peptide was able to compete for the target. NGR exhibited the ability to cross the BBTB and the free peptide was able to compete for the receptor in HUVECs cells **(C, D)**. ^D^WSW- and NGR-modified nanoparticles showed good tumor-targeting in C6 cells **(E, F)**. DiI-positive cells were counted by FCM and intracellular fluorescence was captured by CLSM. Nanoparticles show different targeting capabilities. Scale bars, 10 μm. * indicates P < 0.05.

As shown in **Figure 4A**, the intracellular fluorescence intensity of the NGR-RBCNPs was similar to that of the N-RBCNPs, thus indicating that the NGR-RBCNPs cannot be recognized effectively, and they could therefore evade uptake by bEnd.3 cells. This implies that NGR cannot penetrate the BBB effectively. However, both ^D^WSW-RBCNPs and ^D^WSW/NGR-RBCNPs underwent significant uptake by bEnd.3 cells, indicating that ^D^WSW-modified nanoparticles possess good brain-targeting properties. As shown in **Figure 4B**, untargeted modified nanoparticles were not taken up effectively by cells. We also found that free ^D^WSW (1 mg/mL) significantly inhibited the cellular uptake of ^D^WSW-RBCNPs and ^D^WSW/NGR-RBCNPs (by a factor of 4.5), thus indicating that the ^D^WSW peptide specifically targeted the QS receptor of bEnd.3 cells (21, 53).

Furthermore, to verify the ability of the nanoparticles to target tumor tissues, we measured the cell uptake of NGR-RBCNPs and ^D^WSW/NGR-RBCNPs by using CD13-positive HUVECs. As shown in **Figure 4C**, NGR-RBCNPs and ^D^WSW/NGR-RBCNPs showed stronger intracellular fluorescence than RBCNPs and ^D^WSW-RBCNPs in HUVECs, thus indicating that NGR peptide-modified nanoparticles have good tumor-targeting ability. **Figure 4D** also illustrates the effect of NGR; competition from free NGR (1 mg/mL) for the CD13 receptor led to a reduction in the uptake of NGR-modified nanoparticles (by a factor of 1.5). The rate of uptake for NGR-modified nanoparticles was approximately 1.5 times lower than that of the unmodified nanoparticles. These results indicated that NGR-modified nanoparticles require high expression levels of the CD13 receptor on the target cell in order to function in an appropriate manner. On the other hand, the nanoparticles modified by ^D^WSW peptides showed certain targeting ability, which was consistent with the literature reports (22).

Finally, we investigated the glioma-targeting and uptake ability of the nanoparticles in C6 cells. Compared with unmodified RBCNPs, NGR-RBCNPs, ^D^WSW-RBCNPs, and ^D^WSW/NGR-RBCNPs, were taken up by C6 cells; of these, ^D^WSW/NGR-RBCNPs demonstrated the highest levels of cellular uptake (**Figures 4E, F****)**. The results showed that ^D^WSW and NGR peptide-functionalized RBCNPs have significant tumor-targeting abilities. Overall, these cellular uptake results strongly supported the hypothesis that ^D^WSW and NGR could play a key role in the enhancement of cell recognition and uptake, and the reduction of nonspecific cellular uptake. The data also indicate that ^D^WSW/NGR-RBCNPs would be able to target tumors *in vitro* with reduced levels of non-specific cell uptake. Moreover, as shown in **Figure 4**, preincubation with serum did not impair the cellular uptake of ^D^WSW/NGR-RBCNPs in bEnd.3 cells, HUVECs, and C6 cells, thus indicating that all of the peptide-modified nanoparticles retained their targeting properties in plasma. These results provide a key foundation for further *in vivo* experiments.

#### *In Vitro* Cytotoxicity

We incubated a range of EFL1-loaded nanoparticles, containing different concentrations of EFL1, with C6 cells *in vitro*, and then determined cell survival rate using the MTT assay. As shown in **Supplementary Figure S3D**, increased concentrations of EFL1 were associated with a significant increase in the anti-proliferative activities of free-EFL1 in C6 cells, thus indicating that EFL1 has anti-cancer effects in this type of brain tumor. In addition, all of the formulations containing EFL1 showed cytotoxicity; the largest effect was observed in the free EFL1 group. These results could be explained by the fact that free drugs can rapidly enter cells by passive diffusion under *in vitro* conditions, and with a high concentration gradient. However, nanoparticle-loaded drugs must be released slowly in order to exert their therapeutic effects successfully (**Supplementary Figure S3B**). Accordingly, we found that when compared with free-EFL1, EFL1-loaded biomimetic nanoparticles exhibited a weaker inhibitory effect on the proliferation of C6 cells. These biomimetic nanoparticles can exert important therapeutic effects through targeted modification to enhance the drug concentration delivered to tumor sites. The minor differences in cytotoxicity that were evident between different types of nanoparticles may be related to their uptake by C6 cells and similar patterns of drug release. Further investigation is now needed to investigate these differences further.

#### Transcytosis Efficiency: *In Vitro* BBB and BBTB Models

In order to exert their therapeutic effects for brain disease, it is necessary for drugs to first cross the BBB and then reach a therapeutically relevant concentration. We constructed an *in vitro* BBB model with which to evaluate the penetration effect of nanoparticles with different target modifications. As shown in **Supplementary Figure S4A**, the penetration efficiencies of Cy5.5-labeled ^D^WSW/NGR-RBCNPs (3.22 ± 0.28%) and Cy5.5-labeled ^D^WSW-RBCNPs (3.18 ± 0.32%) were significantly higher than that of Cy5.5-labelled NRG-RBCNPs following a 4 h period of incubation. This showed that the ^D^WSW peptide-modified nanoparticles were highly capable of penetrating the BBB. In addition, to verify the ability of the target-modified nanoparticles to localize to brain tumors, we constructed an *in vitro* BBTB model. Using previously described methods, we successfully established an *in vitro* BBTB model by co-culturing HUVECs/C6 cells and then incubating these co-cultures with various types of RBCNPs (41, 43). As shown in **Supplementary Figure S4B**, the percentage penetration of Cy5.5-labeled ^D^WSW/NGR-RBCNPs (4.09 ± 0.20%), and Cy5.5-labeled NGR-RBCNPs (4.08 ± 0.18%), was significantly higher than that of Cy5.5-labelled ^D^WSW-RBCNPs (1.09 ± 0.23%) after a 4 h period of incubation. This indicated that the NGR peptide-modified nanoparticles exhibited good BBTB penetration. However, NGR-modified nanoparticles must cross the BBB in order to reach the tumor site. These results indicate that the dual-target modified ^D^WSW/NGR-RBCNPs possess the ability to penetrate the BBB and BBTB. These data also confirmed the cellular uptake results (**Figure 4**).

#### Targeting Ability of Biomimetic Nanoparticles in a BBB/C6 Tumor Co-Culture Model

We established a BBB/C6 tumor coculture model to investigate the anti-tumor effect of the target- modified nanoparticles *in vitro*. Once the co-culture model had been established, we used the upper transwell chamber to culture different EFL1-loaded formulations. At the end of the experiment, a sulforhodamine-B staining assay was used to determine the survival rate of C6 cells in the basal chamber. The cell survival rate of C6 cells exposed to free EFL1, EFL1-loaded N-RBCNPs, EFL1-loaded NGR-RBCNPs, EFL1-loaded ^D^WSW-RBCNPs, and EFL1-loaded ^D^WSW/NGR-RBCNPs, was 98.87 ± 13.04%, 97.02 ± 12.36%, 96.88 ± 12.45%, 50.28 ± 5.12%, and 47.02 ± 2.92%, respectively. Results showed that the ^D^WSW peptide-modified nanoparticles could cross the BBB in the *in vitro* model and that the EFL1 drug could be delivered to C6 glioma cells. However, free EFL1, and nanoparticles without ^D^WSW peptide modification, could not pass into the bEnd.3 cells. In contrast with the *in vitro* cytotoxicity testing (**Supplementary Figure S3D**), we found that a single drug could not enter the lesion site in order to provide therapeutic effects, and that drugs require a targeting carrier; in this context, the ^D^WSW peptide is an ideal targeting molecule. The results also showed that the NGR peptide plays a key role when ^D^WSW peptide-modified nanoparticles enter the brain. After passing through the BBB, NGR peptide-modified nanoparticles showed better targeting ability to tumor cells than any of the other preparations. This shows that a dual-target modification strategy was more effective than a single-target modification strategy.

### *In Vitro* Evaluation

#### *In Vivo* Distribution of Biomimetic Nanoparticles

**Figure 5:**
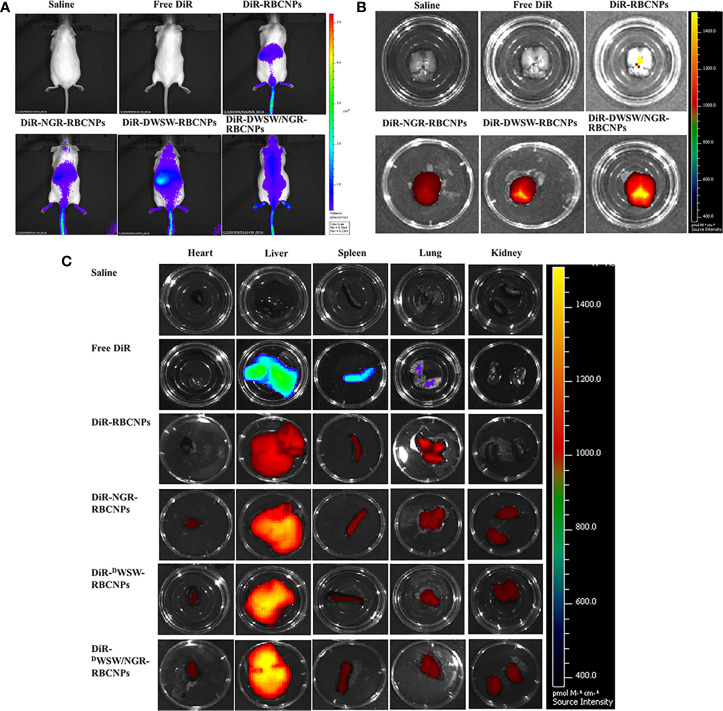
*In vivo* targeting ability test. *In vivo* real-time imaging of different DiR-encapsulated nanoparticles in the brain showing the biodistribution of nanocarriers in animals **(A)**, brain tissue **(B)** and distribution of different DiR-encapsulated nanoparticles in different organs **(C)**. The enhancement of targeted modification leads to increased brain fluorescence. As the liver plays a predominant role in the elimination of these nanoparticles, it was no surprise that the liver showed the highest levels of accumulation when compared to other organs. These data show that these nanoparticles can target drugs to the brain tissue in an effective manner.

Although various anti-tumor drugs are currently available, it is highly evident that the BBB and BBTB create a significant hurdle for drug entry. These important structured makes the clinical treatment of gliomas very challenging. Moreover, because most therapeutic drugs are distributed systemically, they are commonly associated with significant side effects. Therefore, the use of carriers to enable targeted drug delivery to tumor tissue not only increases therapeutic efficacy, but also reduces levels of drug toxicity. To evaluate the brain targeting ability of ^D^WSW/NGR-RBCNPs, we measured whole-body fluorescence signals from intracranial C6 glioma mice. We also investigated the biodistribution of different RBCNPs labeled with DiR within brain tissue. As shown in **Figure 5A**, ^D^WSW-functionalized RBCNPs (^D^WSW-RBCNPs and ^D^WSW/NGR-RBCNPs) were showed good distribution in the brains of experimental mice; the other RBCNP formulations (N-RBCNPs and NGR-RBCNPs) exhibited only minimal brain-targeting ability when compared with controls (basal fluorescence levels). As shown in **Figure 5B**, the distribution of DiR-labelled ^D^WSW/NGR-RBCNPs in brain tissue was more intense than that of DiR-labelled ^D^WSW-RBCNPs, thus suggesting the importance of dual modification. We also investigated the distribution of nanoparticles in other key organs that were harvested at the time of sacrifice. When comparing these other organs, we found that the liver showed the highest nanoparticle content, thus suggesting that the nanoparticles were mainly eliminated by this organ (**Figure 5C**). These results suggest that the ^D^WSW/NGR-RBCNPs could mitigate the toxicity of EFL1.

**Figure 6:**
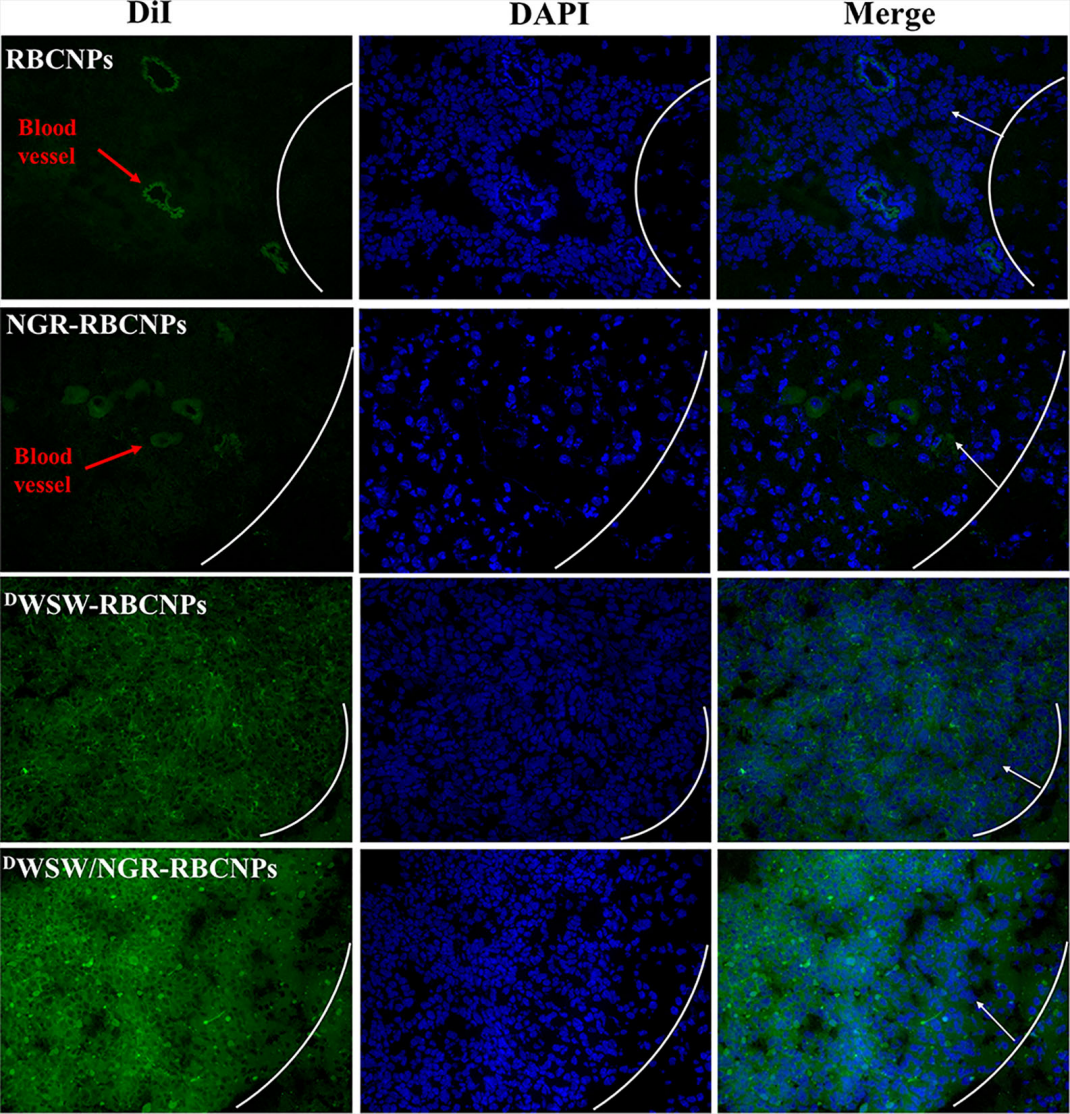
Targeted distribution of nanoparticles in brain tissue. Distribution of DiI-encapsulated nanoparticles in the brain of mice bearing intracranial C6 gliomas, as determined by confocal laser microscopy. The white line shows the margin of the intracranial glioma while the arrows indicate glioma cells. The green color represents DiI-encapsulated nanoparticles, while nuclei are shown in blue (DAPI). 784 DWSW/NGR-RBCNPs showed the strongest targeting ability. Scale bar, 20 μm.

To determine the targeting capability of the ^D^WSW/NGR-RBCNPs to gliomas *in vivo*, we used DiI-labeled RBCNPs to perform immunofluorescence studies on mouse gliomas. As shown in **Figure 6**, unmodified DiI-labeled RBCNPs did not produce fluorescence at the tumor site, thus indicating that the RBCNPs were unable to reach the brain. Only a small number of DiI-labelled NGR-RBCNPs were seen to enter the brain tumor tissue, thus indicating that these nanoparticles were not able to penetrate the BBB effectively. In contrast, we found that DiI-labeled ^D^WSW-RBCNPs were distributed throughout the brain, thus implying that these nanoparticles possessed good levels of ability to penetrate the BBB. DiI-labeled ^D^WSW/NGR-RBCNPs showed stronger levels of fluorescence intensity than DiI-labeled ^D^WSW-RBCNPs in the brain and were also able to enter tumor tissues. This suggests that dual-targeted modified nanoparticles successfully reach tumor tissues after crossing the BBB and BBTB. These results revealed the necessity for dual target modification involving ^D^WSW and NGR.

#### Anti-Glioma Effect *In Vivo*

**Figure 7:**
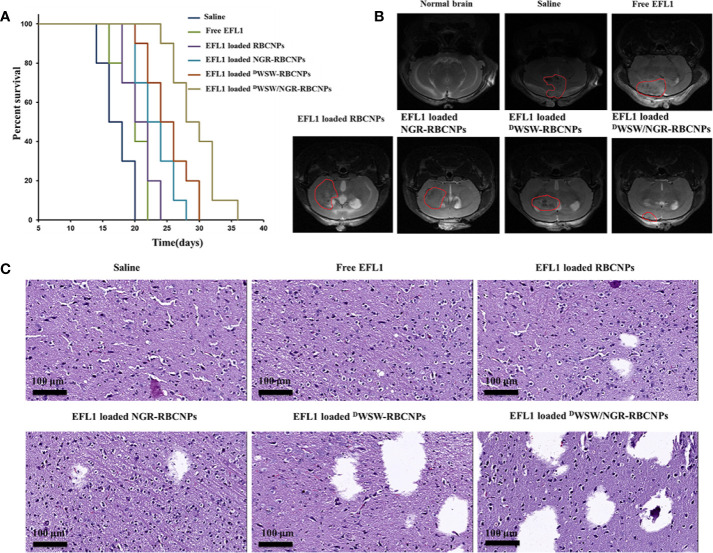
Antitumor effects of nanoparticles *in vivo*. Kaplan-Meier survival curves of nude mice **(A)**, brain MRI images **(B)**, and histological changes in gliomas **(C)** following treatment with different nanoparticles. The strongest therapeutic effect was achieved by the dual-targeted modified nanoparticles.

Using survival time as the main indicator, we performed *in vivo* pharmacodynamic tests on different types of nanoparticles, and used the survival time of each group of mice to plot Kaplan-Meier survival curves. **Figure 7A** shows that treatment with EFL1-loaded ^D^WSW/NGR-RBCNPs achieved the best anti-glioma effect by prolonging the median survival time (36 d); this was 1.8-, 1.6-, 1.5-, 1.3-, and 1.2-fold, longer than that of normal saline, free EFL1, EFL1-loaded N-RBCNPs, EFL1-loaded NGR-RBCNPs, and EFL1-loaded ^D^WSW-RBCNPs, respectively. Because of their inability to penetrate the BBB, the median survival time of the free EFL1 and EFL1-loaded RBCNP groups was similar to that of the normal saline group. Similarly, EFL1-loaded NGR-RBCNPs did not significantly extend the median survival time in the experimental mice. We found that NGR peptide-modified nanocarriers could not transport drugs into the brain tissue. In contrast, ^D^WSW peptide-modified nanocarriers prolonged the survival time of experimental mice. When compared with other groups on day 16, tumor diameter was smallest in the group of mice treated with EFL1-loaded ^D^WSW/NGR-RBCNPs, as determined by MRI (**Figure 7B**). According to previous reports (22), ^D^WSW has the ability to improve the brain-targeting of nanodrug delivery systems. The current results indicate that EFL1-loaded RBCNPs with dual ligand functionalization could significantly improve the treatment efficacy for glioma.

At the end of treatment, we used harvested brain tissue to perform histopathological examinations. Compared with other treatments, the mice treated with EFL1-loaded ^D^WSW/NGR-RBCNPs showed the highest levels of apoptosis (**Figure 7C**). The normal saline group, free EFL1 group, and the EFL1-loaded RBCNP group, showed dense cellular tissues. The data showed that EFL1-loaded ^D^WSW/NGR-RBCNPs were the most effective preparation for the treatment of gliomas.

### Safety Evaluation

Nanoparticles can penetrate into membrane cells and spread along nerve cell synapses, blood and lymphatic vessels. The strong permeability of nanoparticles not only provides effectiveness for the use of drugs, but also poses a potential threat to biological health. In this research, the acute toxicity test in mice was used for safety evaluation. The diet of the experimental mice was maintained at a normal level, and no abnormality in body weight and behavior was observed. The analysis detected no obvious pathological damage in the organs of mice from the saline, free EFL1, and EFL1-loaded ^D^WSW/NGR-RBCNP groups, as shown in **Supplementary Figure S5A**. Furthermore, the three different groups of mice all showed data within the normal range for the following parameters: red blood cell (RBC) count, white blood cell (WBC) count, mean red cell volume (MCV), hemoglobin (HGB), platelet count (PLT), and mean platelet volume (MPV) (**Supplementary Figure S5B**). Further analysis also showed that the following parameters were also within the normal range: alanine aminotransferase (ALT), aspartate aminotransferase (AST), uric acid (UA), triglyceride (TG), high density lipoprotein (HDL), and low-density lipoprotein (LDL) (**Supplementary Figure S5C**). These results indicate that at the experimental doses used herein, the mice showed no obvious abnormalities and no symptoms relating to acute or severe toxicity.

## Discussion

In recent years, biomimetic nano drug delivery system represented by erythrocyte membrane has promoted the development of nanoparticles in the field of nanomedicine and pharmacy (54). Drug molecules can be coupled or complexed with host molecule, adsorbed on the surface, buried under the matrix, or enclosed in the cavity of carriers (55). Commonly used organic nanocarriers include liposomes, solid lipid nanoparticles, nanocapsules, nanospheres, micelles, etc., and inorganic nanocarriers include silica, Fe_3_O_4_, gold nanoparticles, etc. The physicochemical properties of the nanocarrier (size, shape, surface chemical properties, porosity, elasticity, etc.) will affect its biological properties. For example, the size can significantly affect the blood circulation and biological distribution, less than 6 nm is easy to be cleared by the liver, and more than 200 nm is easy to be captured by the liver and spleen. And 30-200 nm nanoparticles can aggregate in tumor site through EPR (enhanced permeability and retention effect) effect. In addition, the shape mainly affects cell phagocytosis, and the surface chemical properties will change the interaction with the physiological environment. Erythrocyte membrane coated nanoparticles have potential applications in many fields (56). First of all, anti-tumor therapy is the most widely studied, including chemotherapy and immunotherapy for glioma, breast cancer, lung cancer and so on. Secondly, inorganic nano carriers were encapsulated for tumor diagnosis and imaging, and the efficiency of photodynamic therapy medium was enhanced to improve its anti-tumor effect. Moreover, nanoparticles prepared by erythrocyte membrane can also be used to treat infectious diseases and autoimmune anemia (57, 58). Erythrocyte membrane is an ideal nanocarrier, and targeted modification enhances its precise treatment of diseases. In the future, it may be widely used in drug delivery, immune regulation, poison adsorption and other fields.

The use of chemotherapeutic approaches for glioma is very challenging. In this study, we developed functional targeted EFL1 nanoparticles in order to deliver drugs to tumor tissue. Historically, the most problematical aspect of delivering drugs into the brain is that most anticancer drugs are unable to penetrate the BBB and BBTB. As a consequence, functional targeted drug nanoparticles are becoming an increasingly attractive option for the treatment of brain cancer. Targeting ligand-modified drug-loaded nanoparticles can improve drug transport through the BBB by recognizing specific receptors that are overexpressed and thus triggering the process of receptor-mediated endocytosis. Of these receptors, the QS receptor is an effective targeting ligand for transporting drugs across the BBB while CD13 can be used as a tumor marker, or tumor cell surface antigen, to transport drugs through the BBTB. In this study, we synthesized DSPE-PEG_2000_-^D^WSW and DSPE-PEG_2000_-NGR conjugates. These were integrated into drug-loaded nanoparticles to transport drugs across the BBB and then target tumor cells. The observed cytotoxicity reflected the levels of apoptosis induced in cancer cells by functional targeted nanoparticles. Transport capacity across the BBB was confirmed by the use of a co-culture model. The analysis of mice with intracranial gliomas clearly demonstrated the accumulation of functional targeted DiR nanoparticles in the brain. The *in vivo* experiments, using mice with intercranial gliomas, confirmed that the targeted DiR nanoparticles exhibited strong antitumor and curative effects, but with minimal levels of toxicity to the experimental animals. As a new drug delivery system, RBCNPs could be used to “disguise” nanoparticles as endogenous substances, thus reducing the risk of recognition by the immune system and uptake by the reticuloendothelial system.

## Conclusions

In the current study, we constructed dual-modified erythrocyte membrane-enveloped PLGA nanoparticles. By modification with ^D^WSW and NGR peptides, the newly developed nanoparticles could be used to deliver drugs to gliomas *via* systemic administration. This drug carrier has two outstanding characteristics: a biomimetic structure and dual targeting capability. ^D^WSW/NGR-RBCNPs were first able to penetrate the BBB and the BBTB, and then target glioma cells. EFL1-loaded ^D^WSW/NGR-RBCNPs significantly improved the efficacy of anti-glioma treatment both *in vitro* and *in vivo*. This research provides a new targeting strategy for the treatment of gliomas. Preliminary validation results showed that NGR and ^D^WSW represent effective ligands for the modification of nano-drug delivery systems. Collectively, the data indicate that ^D^WSW/NGR-RBCNPs have significant potential as a targeted drug delivery system for the treatment of glioma.

## Data Availability Statement

The raw data supporting the conclusions of this article will be made available by the authors, without undue reservation.

## Ethics Statement

The animal study was reviewed and approved by Animal Care and Use Ethics Committee of Beijing Institute of Pharmacology and Toxicology.

## Author Contributions

YC wrote the manuscript and performed the experiments. JS, WH, and MC revised the manuscript. YW, FX, and CG proposed the outline of the article and revised the draft before submission. All authors contributed to the article and approved the submitted version.

## Funding

This research was funded by the Beijing Municipal Natural Science Foundation (grant numbers: 7182097 and 7172162), National Natural Science Foundation of China (grant numbers: 81673597 and 81874305), National Key Research and Development Program of China (grant numbers: 2018YFE0197900 and 2018YFC0115604).

## Conflict of Interest

The authors declare that the research was conducted in the absence of any commercial or financial relationships that could be construed as a potential conflict of interest.
