# Supplementary material for: Dual-Target Peptide-Modified Erythrocyte Membrane-Enveloped PLGA Nanoparticles for the Treatment of Glioma
Source: Front Oncol. 2020 Oct 21;10:563938. doi: 10.3389/fonc.2020.563938 (PMC7609867; doi:10.3389/fonc.2020.563938)
Supplement: Supplementary file 1 [file Table_1.docx]

Supplementary Material

# Supplementary Figures

#
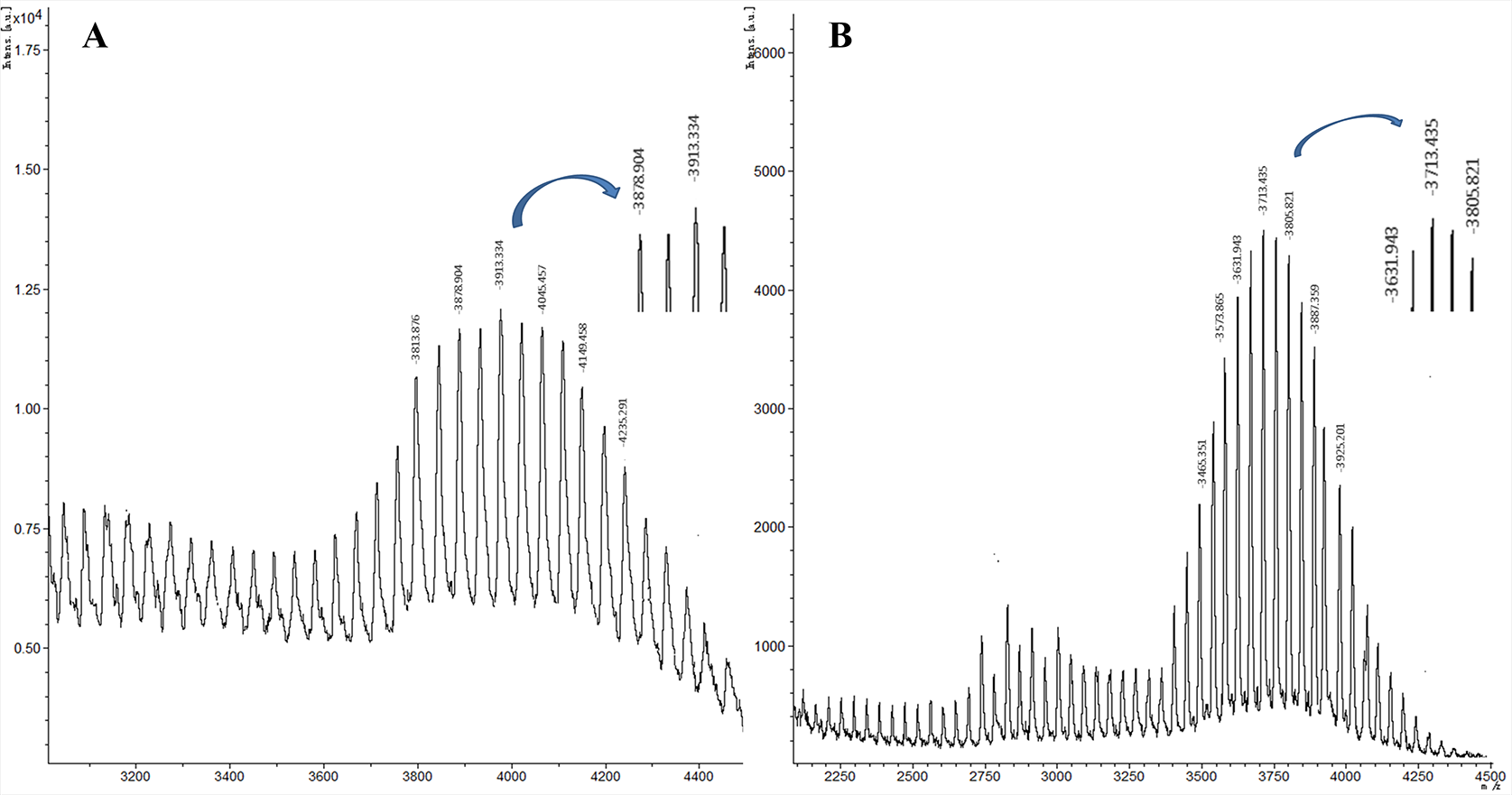


# Supplementary Figure S1. Characterization of targeting molecular materials through MALDI-TOF-MS. DSPE-PEG2000-DWSW (A) and DSPE-PEG2000-NGR conjugate (B).

#
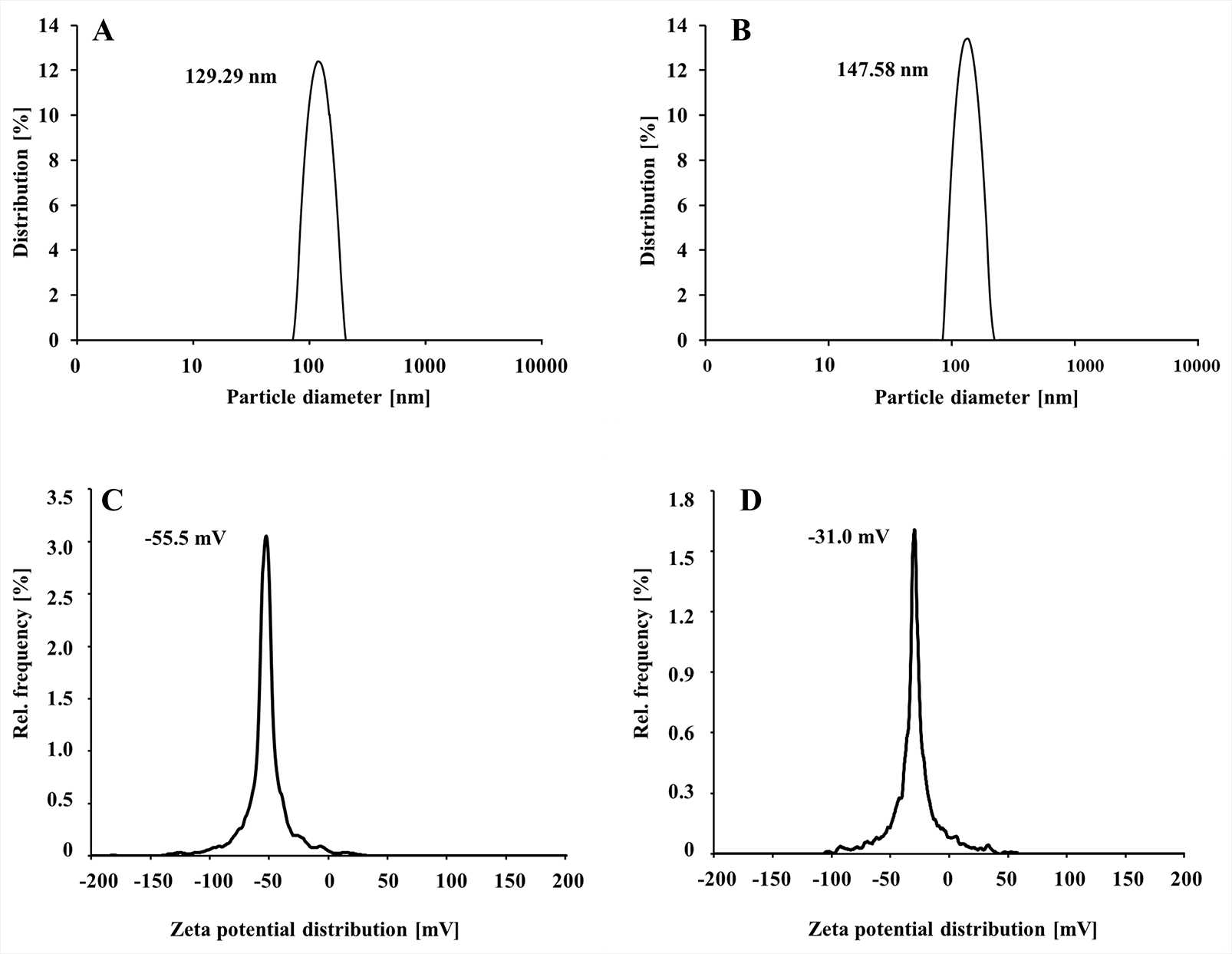


# Supplementary Figure S2. Particle sizes and potential zeta of nanoparticles before and after modification. Particle sizes and zeta potential of PLGA NPs (A, C) and EFL1-loaded DWSW/NGR-RBCNPs (B, D). The particle size increased by approximately 20 nm while the zeta potential decreased by approximately 20 Mv.


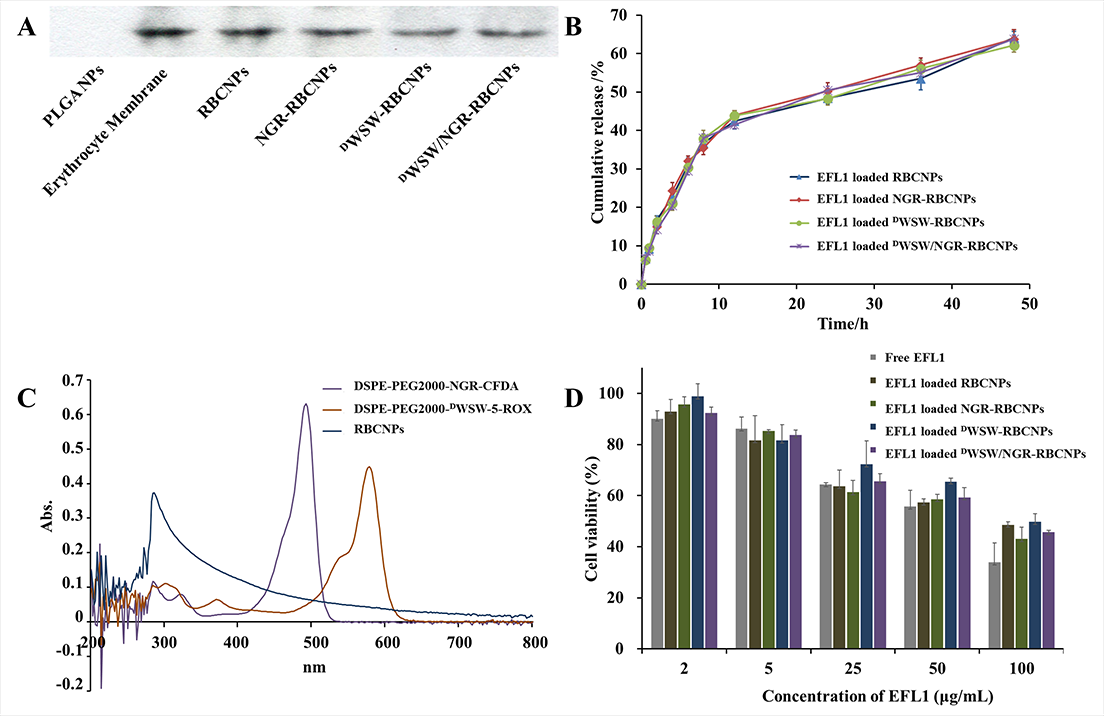


**Supplementary Figure S3.** Physicochemical Property of Nanoparticles. (**A**) CD47 on the surface of erythrocyte membranes. Increased levels of target modification did not influence the properties of erythrocyte membranes. In vitro release of EFL1 from different nanoparticles: (**B**) levels of drug release from different nanoparticles were very similar. (**C**) UV absorption curve of different components showing different absorption peaks. (**D**) *In vitro* cytotoxicity of various EFL1-loaded nanoparticles in C6 cells, as assessed by MTT.


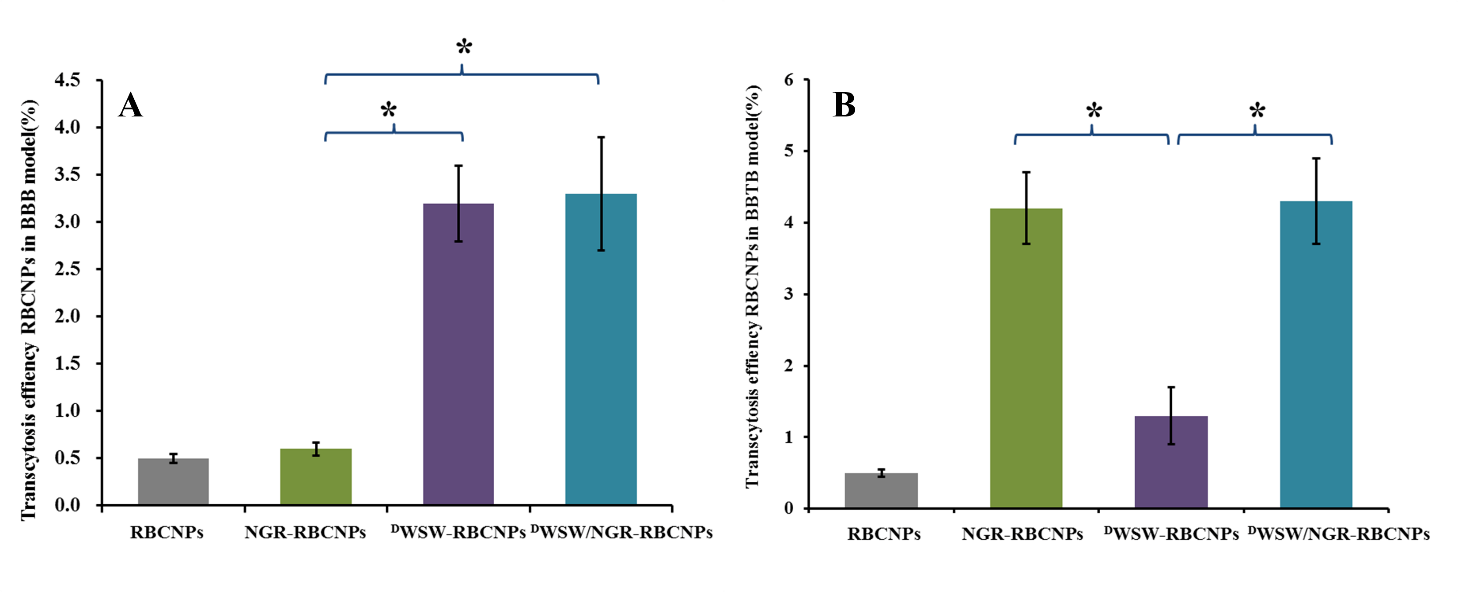


**Supplementary Figure S4.** *In vitro* evaluation of the penetration efficiency of different nanoparticles. bEnd.3/C6 cells were used to establish an *in vitro* BBB model (**A**) and HUVECs/C6 cells were used to establish an *in vitro* BBTB model (**B**). Dual-targeted modified nanoparticles showed the strongest penetrating ability.


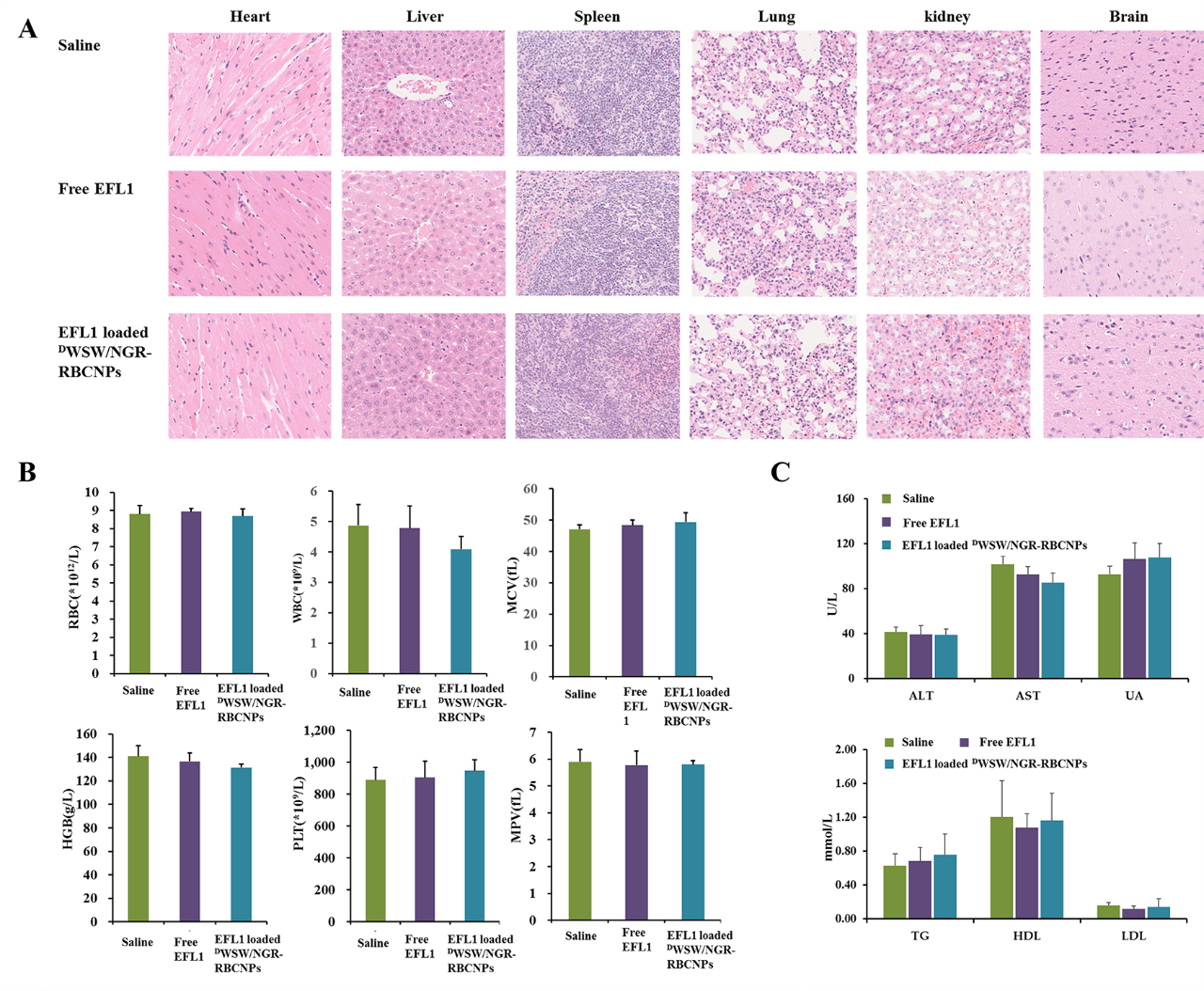


**Supplementary Figure S5.** *In vivo* safety evaluation. (**A**) Histological staining showing that the organs were normal. (**B**) A range of hematological indicators were within the normal range. (**C**) Indices of liver and kidney function were all normal.
